# Respiratory support strategies in neonatal transport in the UK and Ireland

**DOI:** 10.1007/s00431-024-05947-z

**Published:** 2025-01-04

**Authors:** Allan Jenkinson, Theodore Dassios, Nandiran Ratnavel, Anne Greenough

**Affiliations:** 1 Neonatal Transport Service, Royal London Hospital, Bart’s Health NHS Foundation Trust, London, UK; 2 Department of Women and Children’s Health, School of Life Course Sciences, Faculty of Life Sciences and Medicine, King’s College London, Denmark Hill, London, UK; 3 Neonatal Intensive Care Centre, King’s College Hospital, 4Th Floor Golden Jubilee Wing, Denmark Hill, London, SE5 9RS UK

**Keywords:** Ventilation, Neonatal transport, Outcomes

## Abstract

Infants requiring interhospital transfer for a higher level of care in the neonatal period are at increased risk of adverse outcomes. Optimising respiratory management is an important priority. The aim of this survey was to investigate current respiratory support strategies in neonatal transport and identify opportunities for the optimisation of clinical care and future research. A survey of all 18 transport groups in Ireland and the UK was performed. A 10-item structured questionnaire was administered through consultant neonatologists or lead nurses from each transport group between May and June 2024. There was a 100% response rate. There was variation in the types of neonatal ventilator used, and they differed from those on NICUs. A variety of invasive strategies were used, but volume-targeted ventilation was the most common, although different ventilators can deliver different volumes despite apparently the same settings. Non-invasive strategies were used by all, with humidified high flow nasal cannula (HHFNC) being the most common. Continuous carbon dioxide (CO_2_) monitoring was used by most teams (94%): endotracheal CO_2_ assessments by 94% and transcutaneous monitoring by 70%. Only two teams employed closed loop automated oxygen control (CLAC).

*Conclusion*: There is heterogeneity in the ventilators and respiratory strategies used by transport groups. Future research opportunities should include the comparison of those strategies on short- and long‐term outcomes, as well as whether continuous CO_2_ monitoring and CLAC have important benefits.

**Table:** 

**What is Known:** •* Nearly one quarter of neonatal transfers in the UK and Ireland are in infants mechanically ventilated.* •* Optimising respiratory support strategies and reporting respiratory outcomes are research priorities in neonatal transport.*
**What is New:** •* Volume targeted ventilation is the most common respiratory support strategy used in neonatal transport groups in the UK and Ireland, with a heterogeneity of ventilators in use in neonatal transport versus in NICUs.* •* There is a paucity of data reporting respiratory outcomes following neonatal transport including outcomes related to mode of ventilation, continuous carbon dioxide monitoring and closed loop automated oxygen control.*

**What is Known:**

•* Nearly one quarter of neonatal transfers in the UK and Ireland are in infants mechanically ventilated.*

•* Optimising respiratory support strategies and reporting respiratory outcomes are research priorities in neonatal transport.*

**What is New:**

•* Volume targeted ventilation is the most common respiratory support strategy used in neonatal transport groups in the UK and Ireland, with a heterogeneity of ventilators in use in neonatal transport versus in NICUs.*

•* There is a paucity of data reporting respiratory outcomes following neonatal transport including outcomes related to mode of ventilation, continuous carbon dioxide monitoring and closed loop automated oxygen control.*

## Introduction

Neonatal transport groups throughout Europe are responsible for the transfer of vulnerable infants in the neonatal period [1]. Infants requiring interhospital transfer are at increased risk of adverse outcomes including brain injury and death. Furthermore, exposure to neonatal transfer was associated with a higher risk of neurodevelopmental impairment at 3 years of age [2].

The European Standards of Care for Newborn Health states that neonates requiring transport should be transferred by a dedicated, specialised medical service which offers a quality of care similar to that promoted in a neonatal intensive care unit (NICU) [3]. The European Consensus Guidelines on the Management of Respiratory Distress Syndrome emphasise that respiratory best practice standards include lung protective ventilation strategies, and as such, volume-targeted ventilation (VTV) or high-frequency oscillation ventilation (HFOV) should be the first choice for babies with respiratory distress syndrome (RDS) who require mechanical ventilation [4].

The UK Neonatal Transport Group provides uplift, that is for infants transferred from a neonatal unit that does not offer the level of care required, as well as capacity transfers and repatriation. Infants transported are those born prematurely or at term and include those with medical, surgical, cardiac or neurological conditions [5]. The UK-Neonatal Transport Research Collaborative performed a Delphi consensus process which identified respiratory management and outcomes as research priorities in neonatal transport [6]. The aim therefore of this survey was to determine current respiratory support strategies in neonatal transport and identify opportunities to optimise respiratory management and future research.

## Methods

A 10-item structured questionnaire (Appendix) was used. A consultant neonatologist or lead nurse from each transport group in Ireland and the United Kingdom (UK) was contacted via email between May and June 2024. Where a transport group consisted of multiple hubs, a response was sought from each team. Where multiple responses from a transport group were received, follow-up was undertaken to clarify any discrepancies. Subsequently, the teams were contacted to give information on the heating and humidified devices used and the longest distances over which transports occurred.

## Results

**Fig. 1:**
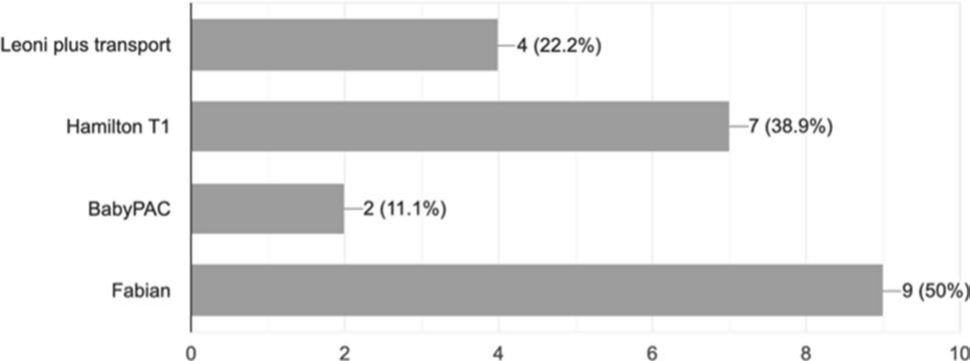
Transport ventilators used in neonatal transport. Four respondents reported use of two ventilators

All 18 transport teams completed the survey with transport consultants being the commonest respondents (77%); the remainder were matrons or lead nurses. The distances infants were transported varied according to the geography in which the team worked in, but the longest distance was 320 km. A variety of ventilators were used (Fig. 1). All groups used invasive ventilation, and ten teams had an invasive ventilation guideline. All teams used pressure and volume-targeted ventilation (VTV) and 12 high-frequency oscillatory ventilation. Twelve teams used volume-targeted ventilation (VTV) as the most common invasive ventilation strategy; five teams used pressure-controlled ventilation as the most common invasive ventilation strategy, and one team did not indicate a preference. All groups reported use of continuous positive airway pressure (CPAP), humidified high flow nasal cannula (HHFNC) and low flow nasal cannula; 11 used bilevel positive airway pressure ventilation, and four used nasal intermittent positive pressure ventilation. The most common non-invasive strategy was HHFNC. Non-invasive support was particularly used for repatriation. Heating and humidification devices included the Fisher and Paykl, Hamilton Humidification and Neopod, with the majority of teams (60%) using the Fisher and Paykl system. Some form of continuous carbon dioxide (CO_2_) monitoring was employed by most teams (94%): endotracheal CO_2_ assessments by 94% and transcutaneous monitoring by 70%. Two teams used closed loop automated oxygen control (CLAC).

## Discussion

We have demonstrated that ventilators used in neonatal transport differ from those used in NICUs. The mechanism of action of the ‘Hamilton T1’ ventilator is via turbine air extraction which is not commonly used in NICU ventilators. Furthermore, the ‘Leoni plus’ has an alternative internal configuration when compared to the ‘Leoni’ used in NICU; the alternative internal configurations reduce shock effects. While the ‘Fabian’ ventilator used in transport is the same as in NICUs, it does not have certification for use in transport. It has not been reported whether the *Fabian* ventilator requires internal configuration changes similar to *Leoni* ventilators to ensure performance during transport. Bench testing has demonstrated that different neonatal ventilators deliver different volumes despite identical ventilator settings, particularly in premature models [7]. Differences in ventilator performance may then result in delays to stabilisation which has been highlighted as a quality metric to assess performance of a specialised neonatal transport programme [8].

VTV was the most commonly used invasive mechanical strategy during neonatal transport. It has been shown to reduce the occurrence of excessive tidal volumes (VTe > 8 ml/kg) and was associated with lower mean VTe compared to pressure-controlled ventilation (4.8 versus 6.0 ml/kg; *p* = 0.0011) during neonatal transport [9]. Excessive volumes in the delivery suite have been associated with intraventricular haemorrhage [10]. Whether they have a similar adverse effect during early transfer of prematurely born infants merits investigation. A retrospective observational study demonstrated that during both HFOV and HFOV-VG, ventilator parameters are maintained close to their targets during transport [11] whether this improves outcomes merits investigation.

In an attempt to reduce the incidence of bronchopulmonary dysplasia (BPD), there has been an increased focus on using non-invasive ventilation, and this has been reflected by changes in neonatal transport respiratory support strategies over the last two decades. In this survey, all transport teams were using non-invasive ventilation with the most common being HHFNC. Randomised trials of HHFNC versus CPAP amongst infants on neonatal units, however, have shown that HHFNC is less successful than CPAP in supporting premature infants [12]. Furthermore, if CPAP fails, there is a higher rate of pneumothorax, longer durations of respiratory support and hospital stay as well as a higher rate of BPD and death [13]. Thus, non-invasive ventilatory strategies during neonatal transport should be evaluated and compared.

There is a paucity of data on the use of capnometry during neonatal transport. Our survey demonstrated that the majority of transfer teams were using continuous CO_2_ monitoring. A UK neonatal transport audit reported variation in rates of hypocapnia (0–10.3%) and hypercapnia (0–9.4%) [5]. Using international transport databases and the UK Neonatal Transport Group data submissions, local and national rates of ventilation and normocapnia were tracked. It was demonstrated that volume-guided ventilation and transcutaneous CO_2_ monitoring had a positive influence on the maintenance of normocapnia during transfer [14]. Whether this influences longer term outcomes needs to be determined.

Two groups reported the use of CLAC using Fabian ventilators. An evaluation of quality metrics to assess performance of neonatal transport programmes included desaturations/cyanosis episodes as one of four physiological parameters to be used [8]. CLAC in the NICU has shown to reduce the incidence and duration of hypoxemic episodes in ventilated infants both those born prematurely and at term. Its use during transport of infants requiring escalation of care thus might improve outcomes.

We designed the survey with a limited number of questions, as we have previously found this resulted in a better response rate. We have, however, subsequently contacted the teams regarding their heating and humidification practices and the longest distances over which they transported infants and included this information. We did not ask the teams if they used particular strategies for particular conditions, as there is no evidence that a particular strategy would benefit infants with certain conditions during transport.

## Conclusion

There was heterogeneity in the ventilators and respiratory strategies used by transport groups, which may reflect the limited evidence base. Future research opportunities should include the comparison of those strategies on short and long‐term outcomes, as well as investigating the possible benefits of continuous CO_2_ monitoring and CLAC. We suggest that a study of closed loop automated oxygen control in ventilated, preterm infants with respiratory disease should be prioritised as we hypothesise that this would reduce clinician manual adjustments during transport and reduce the frequency of hypoxia and hyperoxia.

## Data Availability

No datasets were generated or analysed during the current study.

## Appendix

Questionnaire.

1. Neonatal transport team.

**Table:**

London NTS
KSS – Kent, Surrey, Sussex
PaNDR – East of England
Connect NW
ScotSTAR
NISTAR
Embrace
CenTre
KIDS NTS
SoNAR
SONeT
CHANTS
CHANTS north
NNeTS Newcastle
NNTP

2. Questionnaire respondent:

**Table:**

Consultant
Matron/Lead Nurse

## Ventilation

3. What transport ventilator is used to transport infants on respiratory support?

**Table:**

Leoni plus transport
Hamilton T1
BabyPAC
Fabian
Other (please specify):

4. What non-invasive respiratory support strategies do you have available?

**Table:**

Continuous positive airway pressure (CPAP)
High flow nasal cannula (HFNC)
Low flow nasal cannula
Nasal intermittent positive pressure ventilation (NIPPV)
Bilevel positive airway pressure (BiPAP)
Other (please specify):

5. What is the most common non-invasive respiratory support strategy used?

**Table:**

Continuous positive airway pressure (CPAP)
High flow nasal cannula (HFNC)
Low flow nasal cannula
Nasal intermittent positive pressure ventilation (NIPPV)
Bilevel positive airway pressure (BiPAP)
Other (please specify):

6. Does your transport team have a guideline for invasive ventilation during neonatal transport?

**Table:**

Yes
No

7. What invasive ventilation strategies are available for use in your transport team?

**Table:**

Pressure controlled ventilation (IMV/SIMV/SIPPV/PTV/PSV/PCV)
Volume controlled ventilation (SIMV-VG; VCV; ACV; PVRC; PTV-VC; PCV-VC; S(CMV)+)
High frequency oscillatory ventilation (HFOV)
Other (please specify):

8. What is the most common invasive ventilation strategy used by your transport team?

**Table:**

Pressure controlled ventilation (IMV/SIMV/SIPPV/PTV/PSV/PCV)
Volume controlled ventilation (SIMV-VG; VCV; ACV; PVRC; PTV-VC; PCV-VC; S(CMV)+)
High frequency oscillatory ventilation (HFOV)
Other (please specify):

## Monitoring

9. Is continuous CO_2_ monitoring used during the transport of invasively ventilated infants?

**Table:**

Yes
No

a. If yes, what type of continuous CO_2_ monitoring is performed?

**Table:**

Transcutaneous
Endotracheal

## Closed loop automated control—oxygen

10. Does your transport group used closed loop automated control of oxygen?

**Table:**

Yes
No

## Abbreviations

BPD: Bronchopulmonary dysplasia
CLAC: Closed loop automated oxygen control
CO_2_: Carbon dioxide
CPAP: Continuous positive airway pressure
HHFNC: Humidified high flow nasal cannula
NICU: Neonatal intensive care unit
PCV: Pressure-controlled ventilation
RDS: Respiratory distress syndrome
VTV: Volume-targeted ventilation
